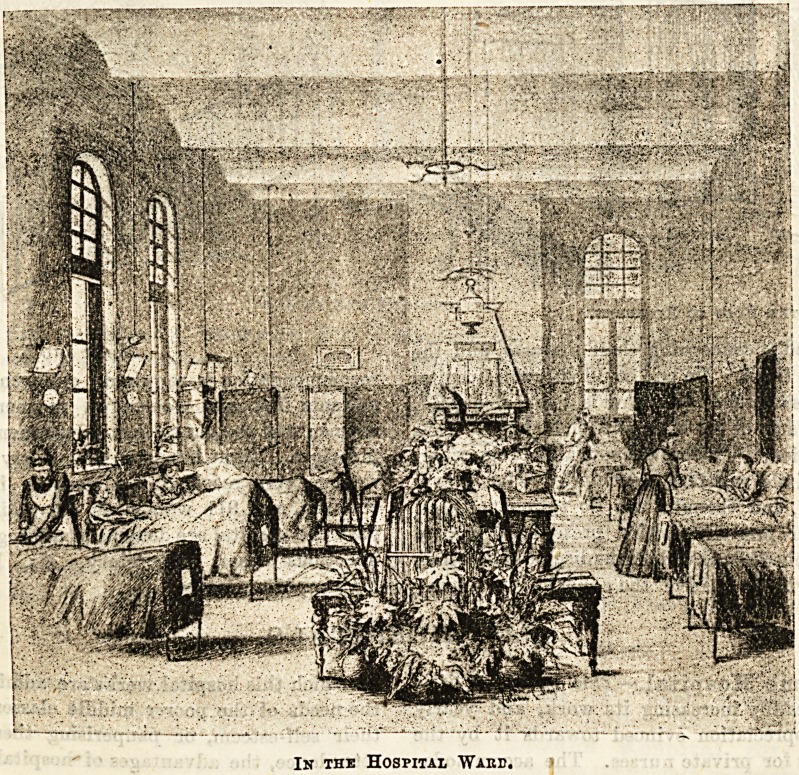# Nervous Diseases, Epilepsy, and Paralysis

**Published:** 1891-01-03

**Authors:** 


### NERVOUS DISEASES, EPILEPSY, AND PARALYSIS.

**National Hospital for Paralysed and Epileptic,** Queen Square, W.C.—This hospital is set apart for the treatment of the most distressing diseases from which a human being can suffer — paralysis and diseases of the nervous system. These cases require the most watchful care and the most advanced medical knowledge, often for a lengthened period before beneficial results may be looked for. The patients come from all parts of the kingdom, and comprise men, women, and children, for paralysis attacks all ages and all ranks, and its results are more dreaded and far-reaching than any other. Of the 210 beds provided in all institutions of the kind in the kingdom, no less than 175 of the total are contained in this National Hospital. One specially benevolent feature of this institution merits the support of all philanthropists, this is the out-pensioners' department for the utterly hopeless and incurable cases, those beyond the power of medical science to benefit, for which this hospital provides pensions, that at least the unfortunate sufferers should not be a helpless burden to their friends. The Committee do all they can to brighten the mind, as well as to alleviate the sufferings of the body, by beautifying the hospital by all means in their power, a power limited, as in all great institutions, by the want of the necessary funds. Secretary, Mr. B. Burford Rawlings, Queen Square, W.C.; Matron, Miss L. C. East

In the Hospital Ward.

**Hospital for Epilepsy and Paralysis,** Portland Terrace, Regent's Park, N.W.—Mr. Howgrave Graham, the secretary, has devoted all his energies to the advancement of this institution, and has achieved a gratifying success. Here, in order to promote providence and to minimise abuse, patients are encouraged to pay what they can afford, but those unable to pay receive free treatment. There is no endowment and no really reliable income, and the hospital has to appeal to the charitable public to aid it in carrying on its really excellent work. Secretary, Mr. Howgrave Graham; Matron, Miss Ridley.

## Figures and Tables

**Figure f1:**